# The Safety of Glycopeptide-Impregnated Calcium Sulphate Following Debridement, Antibiotics and Implant Retention (DAIR) for Infected Total Knee Replacement

**DOI:** 10.7759/cureus.57955

**Published:** 2024-04-10

**Authors:** Richard Barksfield, Pravakar Hamal, Divakar Hamal, Andrew Porteous, James Murray

**Affiliations:** 1 Orthopaedics, Gloucestershire Hospitals NHS Foundation Trust, Gloucester, GBR; 2 Orthopaedics, University Hospital of Llandough, Cardiff, GBR; 3 Intensive Care Unit, Kings College London, London, GBR; 4 Trauma and Orthopaedics, North Bristol NHS Trust, Bristol, GBR

**Keywords:** calcium sulphate, retrospective case-control, safety, debridement implant retention and antibiotics, infected knee arthroplasty

## Abstract

Background and objective

The impact of prosthetic joint infection (PJI) stretches far beyond the physical nature of the disease. It can result in psychological and social consequences, with significant morbidity and mortality for patients. Calcium sulphate-based delivery agents are effective in the management of PJI, yet with associated risks of systemic adverse events.

This study aims to evaluate the risk of systemic adverse events when using calcium-sulphate-based local antibiotic delivery agents in the management of PJIs.

Methodology

We identified 43 patients who underwent debridement, antibiotics and implant retention (DAIR) for infected total knee arthroplasty (TKA) between 2008 and 2014. Patients in the control groupunderwent conventional intravenous and then oral antibiotic administration, while those in the intervention groupunderwent additional local antibiotic therapy via a calcium sulphate alpha hemihydrate matrix. Case notes and laboratory results data were compiled to establish the safety and efficacy of local glycopeptide delivery.

Results

Serum vancomycin levels were within the safe therapeutic range for all patients in the intervention group with no difference in serum assays between treatment groups (intervention 7.7 mg/L; control 8.0 mg/L; *P* = 0.85). Renal function for the study cohort improved at every time point post-operatively when referenced against pre-operative renal function (*P* < 0.05). There was no difference in renal function between intervention and control groups on day 1, one week, six weeks or 12 weeks post-operatively (*P* = 0.78, 0.89, 0.20 and 0.50).

Conclusions

Local glycopeptide delivery via a calcium sulphate alpha hemihydrate matrix did not result in systemic adverse consequences specifically not raising the systemic level of glycopeptide, nor reducing renal function.

Implications for future research

Although demonstrates a safety profile and potential therapeutic benefit, the long-term efficacy of this approach needs to be established. Importantly, selection bias may contribute to masking clinically significant differences in post-operative outcomes.

## Introduction

The incidence of total knee arthroplasty (TKA) is increasing, with US projections anticipating a threefold increase in TKA procedures over the next 15 years, before the global COVID-19 pandemic [1]. Although infection rates following primary TKA are low (0.39%-2%) [2-4], the management of periprosthetic joint infection is costly and results in significant morbidity for the patient. The burden of peri-prosthetic infection will likely rise as the incidence of primary TKA increases and cost-effective management strategies will need to be found.

Accepted rates of infection clearance with debridement, antibiotics and implant retention (DAIR) vary significantly (0%-89%) [5-8], but with sensible case selection, 50% clearance of prosthetic joint infection (PJI) should be achievable [9]. Because of the significant patient morbidity and cost associated with revision TKA, DAIR is a very sensible treatment modality, assuming good case selection [10]. Factors associated with successful DAIR procedures include infection with a low-virulence organism in a healthy host where debridement is performed within four weeks of the onset of infective symptoms [9,11].

Targeted antimicrobial therapy is crucial to the success of DAIR, and traditionally, these have been delivered both locally, using antibiotic-impregnated polymethylmethacrylate cement, and systemically via intravenous (IV) administration. While antibiotic cement (both implant fixation cement and additional beads) is well established in the management of periprosthetic infection, with time, drug elution decreases to a point where antibiotic levels no longer reach minimum inhibitory concentrations, risking the development of antibiotic resistance [12]. In addition, the polymethylmethacrylate (PMMA) beads are non-resorbable, presenting a nidus for residual infection or necessitating a further procedure for removal.

To achieve high local antibiotic elution, without the problems of PMMA beads, we started using an absorbable calcium sulphate local antibiotic delivery agent as an additive technology in our protocol for DAIR of infected TKAs. We selected the cases for this additional local antibiotic delivery as those where we were concerned that standard DAIR may fail. In summary, we chose higher risk cases for additional treatment. In vitro studies of this compound have demonstrated local antibiotic elution above minimum inhibitory concentrations for up to 42 days, but as yet, the systemic effects in vivo have not been quantified [13,14].

## Materials and methods

This was a retrospective case-control study conducted at the Regional Orthopaedic Centre in the United Kingdom. 

Following registration with our local clinical governance department, we identified all patients who had been treated with DAIR for an infected TKA between June 2008 and March 2014 from hospital records and our prospectively collected database. A structured case note analysis was undertaken to establish the clinical course, diagnosis, surgical findings, relevant microbiology and antibiotic regimes. All patients had a confirmed PJI based on consistent organism growth from microbiology specimens, or a single culture-positive sample with features consistent with infection at debridement, for example, the presence of intra-articular pus. Serum vancomycin levels, C-reactive protein (CRP) assays and renal function tests were obtained by interrogation of a regional pathology database. 

Analysis was performed by dividing patients into two groups based on the antimicrobial treatment received: the control group underwent DAIR followed by a traditional combination of IV and then conversion to oral antibiotic therapy; the intervention group underwent DAIR with additional calcium sulphate local antibiotic therapy implanted at the time of their surgical debridement and liner change (Stimulan Rapid Cure, Biocomposites Ltd., Keele, UK). 

At our institution, DAIR is usually performed for infections presenting acutely, with symptoms ideally less than four weeks in duration. Following a routine surgical approach to the knee, five tissue samples are taken for microbiological evaluation, with a further tissue sample sent for histological analysis. An aggressive debridement of all infected or devitalized tissue is then performed with extensive lavage. The retained implants are evaluated for loosening or undermining before the exchange of all modular components, including polyethylene liners and hinge mechanisms where appropriate. In selected cases, locally delivered antibiotic therapy was administered using a calcium sulphate alpha-hemihydrate matrix. The decision to add the additional intra-articular antibiotic delivery was entirely that of the treating surgeon. However, the decision was based on the surgeon's concern that DAIR may not be successful, and consequently, those patients who received additional local antibiotics carried on the calcium sulphate were likely to represent a higher risk of PJI relapse. The matrix was prepared intra-operatively per the manufacturer’s guideline with the addition of antimicrobial agents in a tailored regime for each patient if the microbe was known. Where there was no organism to target, then a broad-spectrum mixture of Vancomycin (1,000 mg powder) and Gentamycin (240 mg solvent) was added to 10 cc of calcium sulphate matrix. The antibiotic-impregnated calcium sulphate beads were then scattered into the knee within the capsule before routine closure with a monofilament suture.

Post-operatively, all patients continued IV antibiotic therapy until confirmation of the infective organism and sensitivities were available from intra-operative samples. A bespoke antibiotic protocol determined by a specialist consultant microbiologist would then be started for each patient based on the virulence and sensitivities of the organism and would include the initial IV agent for two weeks and then an oral antibiotic agent for six months.

Patients underwent routine monitoring of glycopeptide levels, renal function, liver function and inflammatory markers during the post-operative period. Data were assessed at five time points during the treatment protocol: immediately pre-operatively to the DAIR, immediately post-operative to the DAIR, and then at one, six and 12 weeks post-operatively. These serological parameters formed the basis of the primary outcome measures.

Secondary outcome measures included the rate of recurrence of infection and the need for further surgical intervention such as component revision or amputation.

Data were collected and analysed using IBM SPSS Statistics for Windows, Version 28.0 (IBM Corp., Armonk, NY). Normally distributed data were compared using independent samples t-test. Continuous data with a skewed distribution were assessed using a non-parametric Mann-Whitney U test. Categorical data were assessed using the chi-square test or Fisher’s exact analysis where fewer than five observations were made in any group. 

## Results

A total of 43 patients underwent DAIR procedures between June 2008 and March 2014. The median age of those studied was 71.7 years, and there was a slight male preponderance (24/43, 56%; 95% confidence interval [CI] 41%-70%). The median time to follow up was 2.3 years (range 1.0-6.0 years) throughout the whole cohort but was longer in the group that did not undergo treatment with Stimulan Rapid Cure (Stimulan; 3.3 years vs. 1.3 years, *P* < 0.001), and this probably reflects the fact that Stimulan was not in use routinely until early 2012. Demographic data are presented in Table 1.

**Table 1:** Demonstrates analysis of demographic data. ^*^t-test. ^§^Chi-square test. ^^^Fisher’s exact test. ^~^Mann-Whitney U test. DAIR, debridement, antibiotics and implant retention

Variable	Study cohort (*N *= 43)	Intervention (*N *= 14)	Control (*N *= 29)	*P*-value
Age (years)	71.7 (10.6)	73.5 (8.1)	70.9 (11.6)	0.46^*^
Male gender, *n* (%)	24 (56%)	6 (42%)	18 (62%)	0.23^§^
Implantation to DAIR (days)	126 (11-3,864)	126 (12-2,678)	124 (11-3,864)	0.72^~^
Symptoms of DAIR (days)	9 (1-368)	14 (5-368)	6.5 (1-89)	0.046^~^
Plastic surgery involvement, *n* (%)	10 (23%)	5 (36%)	5 (17%)	0.252
Follow-up (years)	2.3 (1.0-6.0)	1.3 (1.0-2.3)	3.3 (1.0-6.0)	<0.001^~^

The median time interval from implantation of the prosthesis to the onset of symptoms of PJI was 4.2 months (range 0.4-129 months), and this was similar in both groups (*P* = 0.72). Those patients in the DAIR plus Stimulan cohort presented with a longer duration of symptoms (14 vs. 6.5 days, *P *= 0.046) than their counterparts treated with standard DAIR alone - this reflected the initial surgical decision to add Stimulan in higher risk cases.

Vancomycin was administered intravenously to 28 (65%, 95% CI 50%-78%) patients overall, and administration was evenly distributed between both treatment groups (*P* = 0.55). The median intravenous dose administered was 1 g, and this ranged between 0.5 and 1.5 g, with no difference in dosage between intervention or control groups (*P* = 0.68). Vancomycin was included in the calcium sulphate matrix for 14 patients (100% of those treated with Stimulan), and 10 of those underwent concurrent intravenous vancomycin therapy with serum assay monitoring. The median vancomycin dose included within the calcium sulphate matrix was 2 g (range 1-4 g). Vancomycin primary assays were performed at a median of 12 hours and 39 minutes following the most recent intravenous dose with no difference in the time interval between intervention and control groups (12 hours 40 minutes and 12 hours 31 minutes, respectively, *P* = 1.00). This was also the case for secondary vancomycin trough assays, performed at a median of 12 hours following the most recent intravenous dose (*P* = 0.69).

**Figure 1:**
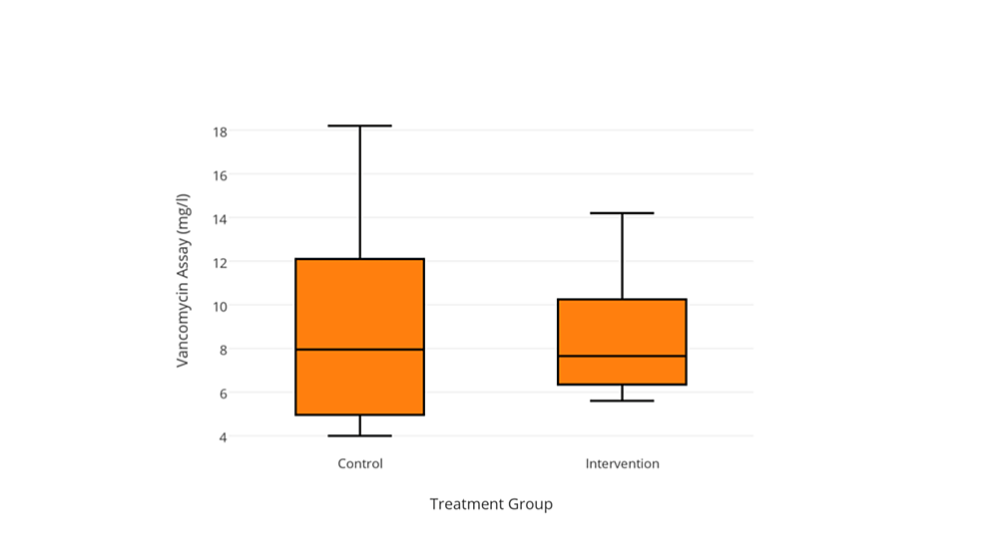
Primary vancomycin trough levels among treatment groups (P = 0.85).

Primary post-operative vancomycin trough levels were recorded within the normal range for all patients in the intervention group with a median level of 7.7 mg/L (range 5.6-14.2), with no discernable difference between control or intervention groups (*P* = 0.85) (Figure 1).

**Figure 2:**
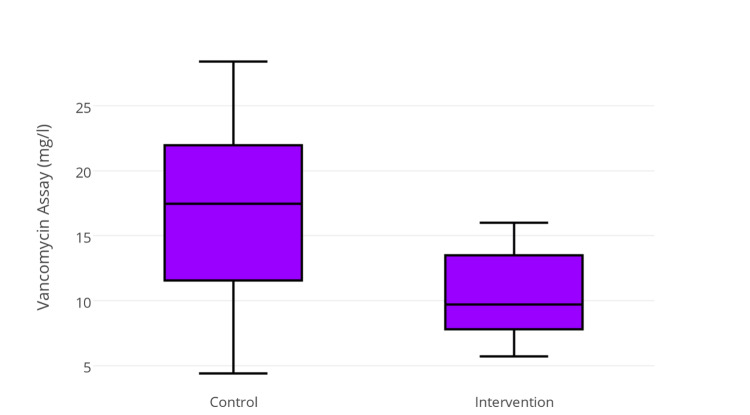
Secondary trough levels among treatment groups (P = 0.037).

One patient in the control group demonstrated high primary trough levels of vancomycin (18.2 mg/L), and this was subsequently discontinued. Secondary trough levels increased significantly (8.0-14.2 mg/L, *P* = 0.004) and were higher in the control group than in the intervention group (17.5 mg/L vs. 10.4 mg/L, *P* = 0.037) (Figure 2).

One patient demonstrated vancomycin levels over the therapeutic range in the intervention group (16 mg/L) compared with five patients in the control group (range 16.7-28.4 mg/L). Vancomycin administration and monitoring data are presented in Table 2.

**Table 2:** Breakdown of glycopeptide administration and serum assays. Post-operative trough interval indicates the interval between DAIR and primary trough level.  1^o^ trough interval represents the time elapsed from the last intravenous vancomycin dose to trough assay. ^§^Chi-square test. ^~^Mann-Whitney U-test. DAIR, debridement, antibiotics and implant retention; IQR, interquartile range

	Study cohort (*N *= 28)	Intervention (*N *= 10)	Control (*N *= 18)	*P*-value
Vancomycin used, *n* (%)	28 (65%)	10 (71%)	18 (62%)	0.55^§^
Vancomycin dose (g)	1.0 (0.5-1.5)	1.0 (0.5-1.5)	1.0 (0.5-1.0)	0.68^~^
Vancomycin in Stimulan mix (g)	-	2.0 [1.0-4.0]	-	-
Post-operative trough interval (days)	2 (IQR 1.0-2.0)	1.5 (1.0-2.0)	2.0 (1.0-2.0)	0.977^~^
1^o^ trough interval (hours:minutes)	12:39 (IQR 9:25-14.34)	12:40 (IQR 9:00-14:29)	12:31 (IQR 9:58-15:23)	1.00^~^
2^o^ trough interval (hours:minutes)	12:00 (IQR 10:29-12:46)	12:08 (IQR 7:30-12:53)	11:40 (IQR 10:34-12:38)	0.69^~^
1^o^ vancomycin trough assay	8.0 (4-18.2)	7.7 (5.6-14.2)	8.0 (4-18.2)	0.85^~^
2^o^ vancomycin trough assay	14.1 (4.4-28.4)	10.4 (5.7-16.0)	17.5 (4.4-28.4)	0.037^~^

**Figure 3:**
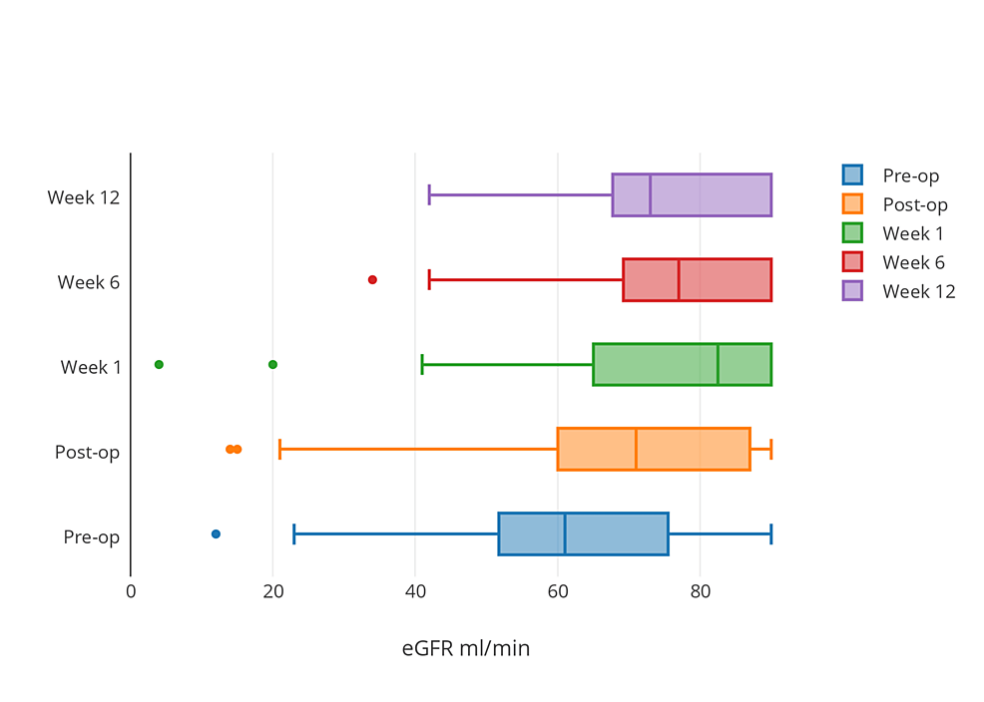
Demonstrates a temporal improvement in estimated glomerular filtration rate (eGFR) among the entire study cohort.

Renal function was deranged in 37 patients (86%, 95% CI 73%-93%) at presentation with a median estimated glomerular filtration rate (eGFR) of 65 mL/minute (range 23-90 mL/minute). Renal function significantly improved between presentation and every subsequent time point post-operatively (*P *< 0.05)(Figure 3).

There was no difference in renal function between intervention or control groups at any point within the timescale of the study (Table 3).

**Table 3:** Evaluation of renal function among treatment groups in the post-operative period. ^~^Mann-Whitney U test. eGFR, estimated glomerular filtration rate

	Study cohort (*N* = 43)	Stimulan group (*N* = 14)	No Stimulan (*N *= 29)	*P*-value
Pre-op eGFR	65 (23-90)	71 (35-75)	57 (34-90)	0.60^~^
Post-op eGFR	79 (15-90)	67 (32-90)	82 (15-90)	0.78^~^
Week 1 eGFR	77 (4-90)	80 (50-90)	73 (4-90)	0.89^~^
Week 6 eGFR	76 (34-90)	74 (42-90)	77 (34-90)	0.20^~^
Week 12 eGFR	80 (42-90)	77 (42-90)	80 (53-90)	0.50^~^

**Figure 4:**
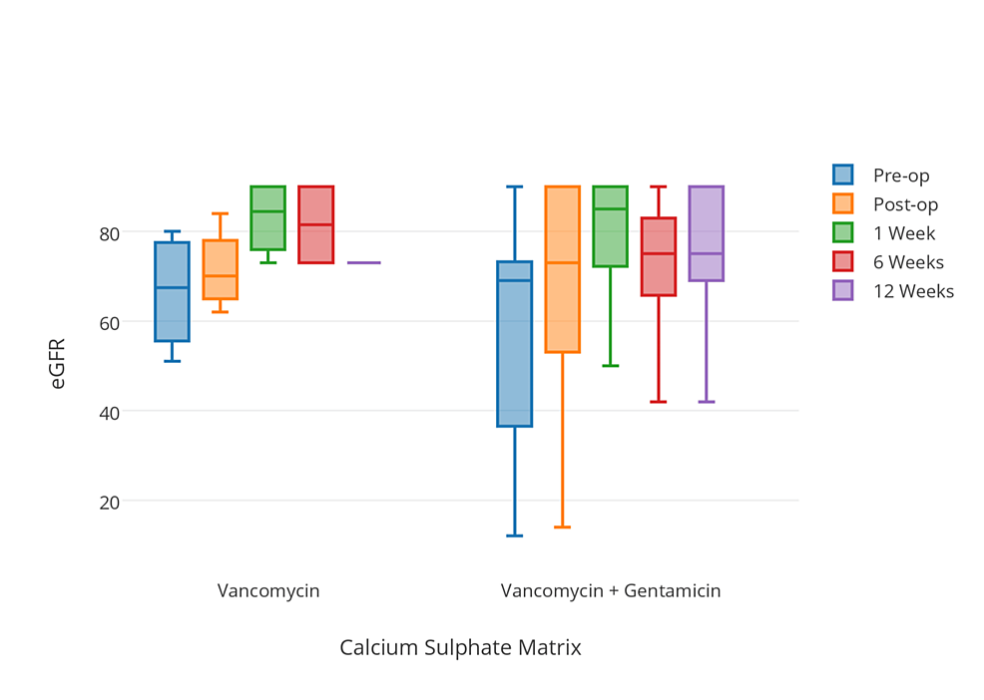
Demonstrates eGFR versus time for patients in the intervention group with two different calcium sulphate antibiotic compositions (P > 0.05 for all time intervals). eGFR, estimated glomerular filtration rate

We did observe a mild reduction in eGFR in the intervention group day 1 post-operatively, but this was not significant (*P* = 0.447) between groups. In addition to local glycopeptide (vancomycin) delivery, an aminoglycoside (Gentamicin) was included in the calcium sulphate matrix for nine patients. Routine monitoring of aminoglycoside levels was not undertaken during the post-operative period, but subgroup analysis of those within the intervention group did not demonstrate any difference in renal function in those receiving additional aminoglycoside therapy (*P* > 0.05 for all time points post-operatively) (Figure 4).

The clinical outcomes for the study cohort are presented in Table 4.

**Table 4:** Outcomes following DAIR for TKR among treatment groups. ^^^Fisher’s exact test. ^~^Mann-Whitney U test. AKA, above-knee amputation; TKR, total knee replacement; DAIR, debridement, antibiotics and implant retention; IQR, interquartile range

	Study cohort (*N *= 43)	Intervention (*N* = 14)	Control (*N *= 29)	*P*-value
Recurrent infection, *n* (%)	11 (26%)	3 (21%)	8 (28%)	0.49^^^
Revision for infection, *n* (%)	9 (21%)	1 (7%)	8 (28%)	0.12^^^
AKA	2 (5%)	2 (14%)	0 (0%)	0.10^^^
Time to 2^o^ procedure (months)	2 (IQR 0.9-7)	1.6 (IQR 1.2-1.8)	2.2 (0.9-12.8)	0.107^~^

Thirty-two patients within the study cohort were treated effectively (74% infection-free survival; 95% CI 60%-85%) at a median follow-up of 2.3 years (range 1.0-6.0). The remaining 11 patients underwent either further revision surgery (9 patients, 21%; 95% CI 12%-36%) or above-knee amputation for intractable infection (2 patients, 4.7%; 95% CI 1.4%-16%). Ten patients required plastic surgical intervention and were treated with debridement and flap coverage (DAIRaF), with five of these in the intervention group (36%; 95% CI 16%-62%) and the remainder in the control group (17%; 95% CI 7.7%-35%). Both amputations occurred within the intervention group and had undergone DAIRaF, with a further patient from this group awaiting two-stage revision. The remaining seven patients who underwent DAIRaF were infection-free at follow-up (70%; 95% CI 39%-89%).

The mortality rate was a striking feature within both groups, in keeping with the increasing knowledge that PJI has a higher rate of mortality than many common cancers. Within the whole cohort, five patients died during the follow-up period (12%; 95% CI 5.1%-25%). Two deaths occurred in the DAIR and Stimulan group (14%; 95% CI 4.3%-40%), and three in the standard DAIR group (10%; 95% CI 3.7%-27%) (*P* = 1.0). No significant differences were observed between intervention and control groups at any point during the post-operative period.

**Figure 5:**
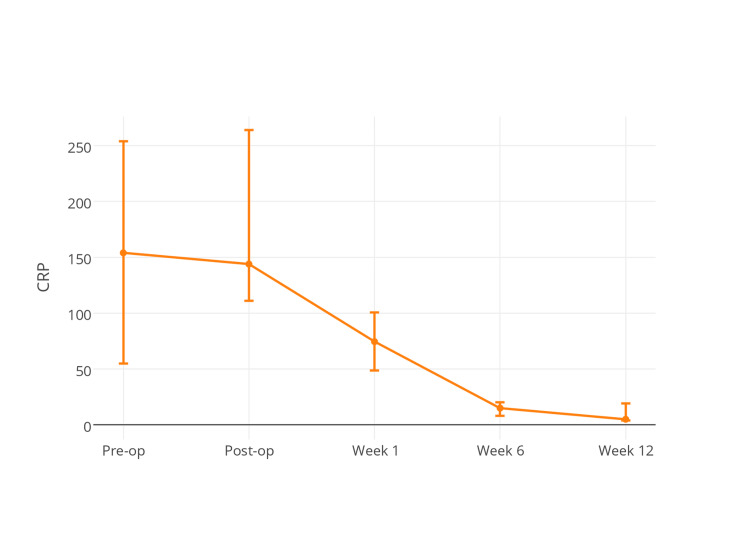
Demonstrates C-reactive protein (CRP) levels among the study cohort with time. No differences were observed between treatment groups at any time point (*P* > 0.05).

C-reactive protein assays demonstrated significant improvement (lowering of the numeric value) between day 1 post-operatively and week 1 (*P* <0.001) and between weeks 1 and 6 post-operatively (*P* < 0.001) throughout the study cohort (Figure 5).

No difference was observed at any time point between the intervention and control groups during the study period (*P* > 0.05 for all measurement intervals). A further subgroup analysis was performed to assess if there was a relationship between CRP and recurrence of infection up to three months following DAIR, but no association was demonstrated using binary logistic regression modelling (*P* > 0.05).

## Discussion

Successful debridement (with modular exchange), antibiotics and retention of infected prostheses (DAIR) have been shown to reduce the costs associated with the management of PJI and improve patient outcomes [3]. This equation needs to be balanced against the risk of a relapse of infection, which leads to further surgical procedures, higher lifetime healthcare costs, reduced patient confidence in their prosthesis and increased patient morbidity, and ranges from 11% to 100% in different series [9]. Improved success rates following DAIR could swing the balance in favour of implant retention over component revision, and naturally, surgeons will search for strategies to improve these outcomes. One such strategy is the local delivery of supraphysiological (systemic) levels of antibiotics through a calcium sulphate matrix, which has been shown to treat biofilm successfully in vitro, but until now, the safety and efficacy of these compounds have not been assessed. We demonstrated that local delivery of vancomycin via a calcium sulphate alpha-hemihydrate matrix was not associated with elevation of systemic vancomycin levels or disturbance of renal function, and this was also the case when gentamicin therapy was included within the calcium sulphate matrix. However, in this selected cohort for additional local delivery in the higher risk cases, we were unable to demonstrate any difference in clinical outcome between patients who received local antibiotic delivery and those who received systemic antibiotic therapy alone. This, of course, is likely due to the selection bias of the cases receiving Stimulan being worse in the opinion of two experienced and recognized PJI knee surgeons. Consequently, this is very promising for the use of this additional local antibiotic matrix for high-risk and potentially all DAIR cases to try and reduce rates of relapse.

This study has several limitations. First, the study was conducted retrospectively and is subject to selection bias in treatment groups. This is most noticeably demonstrated by the difference in follow-up and the duration of symptoms before DAIR procedures between intervention and control groups. We offer two possible explanations for these discrepancies: (1) Calcium sulphate matrices were not available for antibiotic delivery at the start of the period studied and were introduced in early 2012, reducing the follow-up period within the DAIR plus Stimulan cohort. (2)Patients presenting with a longer duration of symptoms are known to be at higher risk of failure of DAIR procedures. In cases where the decision to attempt a DAIR has been made, perhaps in high comorbidity where the patient is less ideal for extraction of implants, then a *salvage strategy* is used, where every possible treatment modality is employed to improve the chance of success despite a compromised starting position. This may explain why two amputations occurred in the intervention group, while there was a trend towards an increased number of secondary revision procedures in the control group. Any future work should seek to address this inbuilt bias with prospective randomization and stratification for known confounders.

Second, the duration of follow-up was relatively short, with a median of 2.3 years for the entire cohort and was shorter still in the intervention group, with a median of 1.3 years. On closer inspection of the results, however, it was apparent that the majority of adverse outcomes occurred in the early post-operative period at a median of two months following the DAIR procedure, while 75% of recurrences of infection occurred within seven months. It is possible, therefore, that the figures presented underestimate the risk of failure of DAIR procedures, but we would suggest that these effects would be relatively modest and unlikely to distort the results of the study dramatically.

Third, the obvious criticism of a study that fails to demonstrate a difference between treatment groups is whether or not the study is adequately powered to detect such differences. However, the main aim of this study is to assess the effects of local high-concentration glycopeptide therapy on systemic glycopeptide level and host renal function within a pragmatic clinical setting for knee PJI. Based on our results, a post hoc sample size calculation suggested that to demonstrate a clinically important difference in serum glycopeptide levels (e.g., 5 mg/L) with a study powered to 80% and alpha error of <0.05, then seven participants in each treatment group would be required. Therefore, while the number of patients in the present study is relatively small, we do believe it is sufficiently powered to demonstrate a clinically important difference in our primary endpoint.

Finally, while our primary interest was in the systemic effects of local and intravenous (IV) vancomycin therapy, several heterogeneous antibiotic regimes were used in both the peri-operative and post-operative periods. There were no common trends in the antibiotic combinations used, due to the range of infecting organisms, and hence, a formal analysis of treatment effects was not possible. We recognise that this represents a significant confounding variable but would be very difficult to control without prospective randomisation and formalised study protocols. Future research could aim to address the issue of heterogeneous regimes by clear documentation of dose and duration intra-operatively and post-operatively.

All patients undergoing DAIR procedures with concurrent IV and local calcium sulphate glycopeptide delivery demonstrated primary serum vancomycin levels within the safe therapeutic window in the immediate post-operative period. It is interesting that secondary trough levels were higher than primary trough levels in both treatment groups and were excessively raised in the control group. We do not have clear data that explains this discrepancy. None of the vancomycin doses increased following primary trough levels, and we propose that these effects are possibly the result of antibiotic accumulation due to incomplete renal excretion, with the return to normal renal clearance being slower in the standard DAIR rather than the DAIR with the Stimulan group. 

An in vitro study examined the elution of vancomycin from calcium sulphate alpha hemihydrate matrices and demonstrated that peak concentrations occurred within 48 hours of administration, while local vancomycin levels remained therapeutic up to 42 days [13]. Within the intervention group of the present study, primary vancomycin assays were performed a median of 1.5 days following the administration of vancomycin-impregnated calcium sulphate beads. While this is not optimal timing for capturing the peak of calcium sulphate vancomycin elution, the time-elution analysis from the in vitro study suggests that levels taken at 36 hours should be approaching peak levels on the upswing of the time-elution graph [13]. We, therefore, believe our findings are a realistic representation of the in vivo pharmacokinetics of vancomycin-impregnated calcium sulphate beads.

Renal function was deranged in 86% of patients at presentation and serves as a reminder of the toxic systemic effects of acute PJI. Reassuringly, there was no difference in renal function between those patients treated with combined IV and local vancomycin therapy and those treated with IV therapy alone at any time point. Furthermore, in the patients who underwent concurrent local gentamicin therapy, no difference in renal profile was demonstrated when compared to vancomycin alone.

We are not aware of any literature that has specifically examined nephrotoxicity or systemic antibiotic elution following implantation of vancomycin-impregnated calcium sulphate beads for orthopaedic infections. Nephrotoxicity has, however, been a concern in many studies examining the elution of tobramycin from calcium sulphate matrices. Humm et al. reported on the use of tobramycin-impregnated beads for the management of tibial osteomyelitis and identified that one of the 21 patients treated sustained an acute kidney injury, although subsequently this was thought more likely due to pre-existing nephropathy and IV antibiotic administration [15]. A further in vivo assessment of tobramycin elution from an alternative calcium sulphate matrix demonstrated a transitory peak above safe tobramycin serum levels at 12 hours, before reaching safe levels at 24 hours, and cautioned against use in those with significant degrees of renal failure [16]. 

Concerns regarding the use of high-dose glycopeptides are well-founded. Although modern preparations of vancomycin are thought to have fewer nephrotoxic effects, Lodise et al. demonstrated a strong relationship between initial trough levels and the nephrotoxic effects of IV vancomycin therapy, with levels greater than 20 mg/L associated with a 33% rate of nephrotoxicity [17]. Vancomycin therapy delivered locally via a calcium sulphate matrix may have significant advantages over systemic administration with prolonged elution at levels over the minimum inhibitory concentration [13] with a low risk of systemic toxicity, as we have demonstrated.

While we were unable to demonstrate any difference in the outcome of those managed with combined systemic and locally delivered glycopeptide therapy versus those receiving systemic administered glycopeptides alone, we suggest this is likely due to selection bias, with the additional local antibiotic matrix being used in the higher risk cases. Overall, however, we report infection-free survival in 74% of patients at a median 2.3-year follow-up, which compares favourably with other contemporaneous series presented in Table 5.

**Table 5:** Success rates following DAIR procedures in the recent literature. DAIR, debridement, antibiotics and implant retention; I + D, irrigation and debridement; DECRA, debridement modular exchange, component retention and parenteral antibiotic

Author	Year	Participants	Procedure	Mean follow-up period (Years)	Success rate
Byren et al. [5]	2009	112 joints: 52 hip, 51 knee, 9 other	DAIR	2.3	82%
Cobo et al. [6]	2010	117 joints: 69 hip, 53 knee, 17 other	DAIR	2.0	70.1%
Van Kleunen et al. [8]	2010	18 joints: 13 hip, 5 knee	DAIR	2.6	72%
Koyonos et al. [7]	2011	138 joints: 60 hip, 78 knee	I + D (Liner not exchanged)	4.5	35%
Odum et al. [11]	2011	150 joints: 53 hip, 97 knee	I + D (Liner exchanged)	Not stated	31%
McPherson et al. [14]	2013	24 joints: 8 hip, 16 knee	DECRA	Not stated	88%
This study	2015	43 knee	DAIR	2.3	74%

## Conclusions

Local glycopeptide delivery via a calcium sulphate alpha hemihydrate matrix does not adversely affect systemic glycopeptide levels or impair renal function in patients undergoing DAIR for infected total knee replacement (TKR). The long-term efficacy of this approach needs to be established. In view of the potential benefit of ultra-high local antibiotic concentrations and the safety profile we have demonstrated, we propose the use of this antibiotic delivery system should be encouraged as a promising technique.

## References

[1] Kurtz S, Ong K, Lau E, Mowat F, Halpern M (2007). Projections of primary and revision hip and knee arthroplasty in the United States from 2005 to 2030. J Bone Joint Surg Am.

[2] Blom AW, Brown J, Taylor AH, Pattison G, Whitehouse S, Bannister GC (2004). Infection after total knee arthroplasty. J Bone Joint Surg Br.

[3] Garvin KL, Konigsberg BS (2011). Infection following total knee arthroplasty: prevention and management. J Bone Joint Surg Am.

[4] Peersman G, Laskin R, Davis J, Peterson M (2001). Infection in total knee replacement: a retrospective review of 6489 total knee replacements. Clin Orthop Relat Res.

[5] Byren I, Bejon P, Atkins BL (2009). One hundred and twelve infected arthroplasties treated with 'DAIR' (debridement, antibiotics and implant retention): antibiotic duration and outcome. J Antimicrob Chemother.

[6] Cobo J, Miguel LG, Euba G (2011). Early prosthetic joint infection: outcomes with debridement and implant retention followed by antibiotic therapy. Clin Microbiol Infect.

[7] Koyonos L, Zmistowski B, Della Valle CJ, Parvizi J (2011). Infection control rate of irrigation and débridement for periprosthetic joint infection. Clin Orthop Relat Res.

[8] Van Kleunen JP, Knox D, Garino JP, Lee GC (2010). Irrigation and débridement and prosthesis retention for treating acute periprosthetic infections. Clin Orthop Relat Res.

[9] Parvizi J, Cavanaugh PK, Diaz-Ledezma C (2013). Periprosthetic knee infection: ten strategies that work. Knee Surg Relat Res.

[10] Kallala RF, Vanhegan IS, Ibrahim MS, Sarmah S, Haddad FS (2015). Financial analysis of revision knee surgery based on NHS tariffs and hospital costs: does it pay to provide a revision service?. Bone Joint J.

[11] Odum SM, Fehring TK, Lombardi AV (2011). Irrigation and debridement for periprosthetic infections: does the organism matter?. J Arthroplasty.

[12] van de Belt H, Neut D, Schenk W, van Horn JR, van der Mei HC, Busscher HJ (2001). Infection of orthopedic implants and the use of antibiotic-loaded bone cements. A review. Acta Orthop Scand.

[13] Aiken SS, Cooper JJ, Florance H, Robinson MT, Michell S (2015). Local release of antibiotics for surgical site infection management using high-purity calcium sulfate: an in vitro elution study. Surg Infect (Larchmt).

[14] McPherson MFE, Matthew Dipane BA, Sherif Sherif MD (2013). Dissolvable antibiotic beads in treatment of periprosthetic joint infection and revision arthroplasty - the use of synthetic pure calcium sulfate (Stimulan®) impregnated with vancomycin & tobramycin. Reconstr Rev.

[15] Humm G, Noor S, Bridgeman P, David M, Bose D (2014). Adjuvant treatment of chronic osteomyelitis of the tibia following exogenous trauma using OSTEOSET(®)-T: a review of 21 patients in a regional trauma centre. Strategies Trauma Limb Reconstr.

[16] Livio F, Wahl P, Csajka C, Gautier E, Buclin T (2014). Tobramycin exposure from active calcium sulfate bone graft substitute. BMC Pharmacol Toxicol.

[17] Lodise TP, Patel N, Lomaestro BM, Rodvold KA, Drusano GL (2009). Relationship between initial vancomycin concentration-time profile and nephrotoxicity among hospitalized patients. Clin Infect Dis.

